# Growth, carcass traits, cecal microbial counts, and blood chemistry of meat-type quail fed diets supplemented with humic acid and black cumin seeds

**DOI:** 10.5713/ajas.18.0148

**Published:** 2018-05-31

**Authors:** Muhammad Arif, Abdur Rehman, Mohamed E. Abd El-Hack, Muhammad Saeed, Fateh Khan, Muhammad Akhtar, Ayman A. Swelum, Islam M. Saadeldin, Abdullah N. Alowaimer

**Affiliations:** 1 Department of Animal Sciences, College of Agriculture, University of Sargodha, Punjab 40100, Pakistan; 2 Department of Poultry, Faculty of Agriculture, Zagazig University, Zagazig 44511, Egypt; 3 Institute of Animal Sciences, Faculty of Animal Husbandry, University of Agriculture, Faisalabad 38040, Pakistan; 4 CVSD Remount Depot Sargodha Cantt, Punjab 40100, Pakistan; 5 Department of Animal Production, College of Food and Agriculture Sciences, King Saud University, P.O. Box 2460, Riyadh 11451, Saudi Arabia; 6 Department of Theriogenology, Faculty of Veterinary Medicine, Zagazig University, Zagazig 44511, Egypt; 7 Department of Physiology, Faculty of Veterinary Medicine, Zagazig University, Zagazig 44511, Egypt

**Keywords:** Black Cumin, Blood, Carcass, Humic Acid, Microbial, Performance, Quail

## Abstract

**Objective:**

The present study attempted to determine safe and sufficient growth promoters in poultry feeding.

**Methods:**

A total of 520 seven-day-old quail chicks were randomly allotted to eight treatment groups in a 4×2 factorial design experiment to evaluate the effect of different levels of humic acid (HA) and black cumin (BC) seed and their interactions on growth, carcass traits, gut microbes, and blood chemistry of growing quails. Quails were randomly distributed into 8 groups in a 4×2 factorial design, included 4 HA levels (0, 0.75, 1.5, and 2.25 g/kg diet) and 2 BC levels (0 or 5 g/ kg diet).

**Results:**

Increasing HA level associated with a gradual increase in final weight, feed intake and body weight gain along with an improvement in feed conversion ratio. Dietary addition of 5 g BC powder/kg diet gave similar results. The highest level of HA (2.25 g/kg diet) recorded the best values of carcass weight, breast yield, intestinal length, and intestinal weight comparing with the control and other HA levels. Total viable microbial counts decreased (p<0.05) with increasing levels of HA except the intermediate level (1.5 g/kg diet). The concentration of serum cholesterol and low density lipoprotein (excluding that 0.75 g HA) decreased (p<0.05) and high density lipoprotein increased (p = 0.034) along with increasing HA level. The interaction between the 2.25 g HA×5 g gave the best results regarding most studied parameters.

**Conclusion:**

These findings indicated that HA combined with BC could be used as effective growth promoters, with the recommended level being 2.25 g HA+5 g BC/kg of quail diet.

## INTRODUCTION

Different antimicrobial additives are used worldwide in poultry feed to improve dressing percentages and growth rates, and to reduce disease effects [1]. Feed additives other than antibiotics are preferred nowadays as antibiotics are banned in most countries especially in the European Union. The residual effect of antibiotics in poultry products develops resistance against bacteria and therefore minimizes its medicinal effect in humans [2]. To overcome this, some additives such as plant extracts and organic acids are being tested as alternatives to antibiotics [3].

Researchers have observed that humic acid (HA)-based mixtures have the ability to be an alternative to antibiotic growth promoters in broiler diets [2]. The HA is a product of the decomposition of organic matter, particularly that of plant origin. The use of humates in animal nutrition as feed additives was a new idea and particular humates have been used for disturbances of the digestive system including diarrhea and malnutrition as a component of replacement therapy [4]. Better feed efficiency with minor mortality has been reported in broilers utilizing humates [2]. Previous experimental studies have indicated HA to be non-toxic and non-teratogenic [3].

Aromatic plants and extracts have become more applicable because of their antimicrobial and stimulating impacts on the digestive system of animals [5]. As an aromatic plant, black cumin (BC) or *Nigella sativa* is grown widely in various parts of the world and its seeds have been used to enhance human health in many countries especially in Southeast Asia and the Middle East. Many studies have indicated that BC could improve the productive performance of poultry [6–8]. In some broiler studies, it was reported that BC seeds had greater effects on weight gain and feed conversion ratio (FCR) [9].

Both HA and BC possess the potential to promote quail growth performance by reducing bacterial and mold growth, thus reducing toxin levels [10]. The hypothesis of the present study was that the two additives (HA and BC seed) would reveal a synergistic action. Limited information is available regarding the use of HA and BC in combination as feed additives for poultry. Therefore, the aim of the present study was to investigate the impact of the combination of HA and BC seed on growth performance, carcass traits, and blood chemistry of meat-type quails.

## MATERIALS AND METHODS

### Ethical statement

The present experiment was conducted at the Poultry Research Station, University College of Agriculture, University of Sargodha, Sargodha, Pakistan. All experimental procedures were carried out according to the Local Experimental Animal Care Committee and approved by the Ethics of the Institutional Committee at the University College of Agriculture, University of Sargodha. The total duration of the experimental trial was 35 days.

### Birds, management, and diets

A total of 520 seven-day-old unsexed quail chicks were randomly allotted to 8 treatment groups of 5 replicates (13 birds/replicate) in a 4×2 factorial design experiment. Chicks were housed in wooden cages (40 cm high×40 cm width×40 cm length). Feed and water were offered *ad libitum* throughout the experimental period. The birds were reared in *iso*-managerial, hygienic, and environmental conditions. Light was provided for 23 h a day. Indoor temperature was around 27°C and the average relative humidity was 50%.

Eight *iso*-caloric and *iso*-nitrogenous diets were formulated to cover the nutrient requirements for growing Japanese quail based on the protocol by NRC [11]. Quails were randomly distributed into 8 groups in a 4×2 factorial design, included 4 HA levels (0, 0.75, 1.5, and 2.25 g/kg diet) and 2 BC levels (0 or 5 g/kg diet). Composition and chemical analysis of the experimental diets are shown in Table 1.

The HA and BC seeds were purchased from Faisal Traders (Sargodha, Pakistan). The chemical composition (%) of BC used in the present study was as follows: dry matter (DM) 92.54; organic matter (OM), 94.54; ash, 5.46; crude protein (CP), 32.84; ether extract (EE), 17.11; crude fiber (CF), 15.93; and nitrogen free extract, 29.78.

### Investigated measurements

#### Growth performance

Individual live body weight was recorded at 1, 3, and 6 weeks of age. Body weight gain was calculated during the experimental period. Feed intake was recorded weekly on a replicate basis to estimate FCR as feed intake (g)/weight gain (g). Birds were observed twice daily for mortality, which was zero.

#### Carcass characteristics

At the end of the experiment (6 weeks of age), 40 female birds (5 from each treatment group) were slaughtered and sampled for carcass evaluation. The carcasses were weighed, and the weights of the liver, gizzard, and heart were recorded and expressed as percentage of slaughter weight.

#### Bacterial count

At the end of the trial, 2 birds from each replicate were euthanized and the cecum was removed for further culture. Total counts of coliform, *Escherichia coli* (*E. coli*), and *Clostridium perfringens* were measured by membrane filter techniques following the method described by Brantner et al [12]. The cecum pH was determined using a digital pH meter (MP511 Benchtop pH Meter; Apera Instruments).

#### Blood sampling

Blood samples were randomly collected from 5 birds per treatment after euthanasia into sterilized tubes that were closed with rubber stoppers. Samples were left to coagulate and centrifuged at 3,500 rpm for 15 min to obtain serum, and the serum samples were stored in Eppendorf tubes at −20°C until analyzed. The following serum biochemical parameters: alkaline phosphate (μ/L), alanine amino transferase (μ/L), aspartate amino transferase (μ/L), glutathione peroxidase (U/L), superoxide dismutase (U/L), cholesterol (mg/dL), triglyceride (g/dL), high density lipoprotein (HDL, mg/dL), low density lipoprotein (LDL, mg/dL), calcium (mg/dL), and phosphorus (mg/dL) concentrations were determined spectrophotometrically using commercial diagnostic kits from Faisal Traders (Sargodha, Pakistan) based on procedures described by Akiba et al [13]. Glucose concentration was analyzed following the technique described by Malheiros et al [14].

#### Feed and black cumin samples

Proximate analysis of tested BC (*Nigella sativa*) and basal diets were performed based on the procedures described by AOAC [15] for determination of DM (ID 930.15); OM (ID 942.05); CP (ID 954.01); EE (ID 945.16); and CF (ID 978.10).

### Statistical analysis

Data were analyzed using general liner model procedure in SPSS [16]. A 4×2 factorial arrangement was used to analyze data of all parameters. The model utilized included the impacts of HA and BC, as well as the interaction impacts:

$${\text{Y}}_{\text{ij}}=\mu +{\text{HA}}_{\text{i}}+{\text{BC}}_{\text{j}}+{\text{HABC}}_{\text{ij}}+{\text{e}}_{\text{ij}}$$

Where, Y_ij_ = an observation, μ = the overall mean, HA_i_ = fixed impact of humic acid levels (i = 0, 0.75, 1.50, and 2.25 g/kg diet), BC_j_ = fixed impact of black cumin powder levels (j = 0, 5.0 g/kg diet), HABC_ij_ = fixed impact of interaction between HA and BC levels (j = 1, 2, ..., and 8) and e_ij_ = random error associated to each observation. The differences among means were determined using the post-hoc Newman-Keuls test (p<0.05).

## RESULTS AND DISCUSSION

### Growth performance

Results presented in Table 2 illustrate the impact of dietary treatments on growth performance traits. Excluding initial body weight, all growth performance parameters were significantly (p>0.05 or 0.01) altered. It is obvious that increasing HA level associated with a gradual increase in final weight, feed intake and body weight gain along with an improvement in FCR. Our results are similar to the findings of Mozafar et al [17] who discovered that the addition of HA in feed increased body weight gain of chicks. This result may be explained by the fact that HA increases animals’ resistance versus stress factors like heat, which positively reflects on the animal performance [18]. It has also been stated that the gut microflora of animals is stabilized by HA, which results in improved nutrient absorption and increased weight gain [10]. The HA forms a protective film on the mucous epithelia of the gastro-intestinal tract against infections and toxins [2]. The macro-colloidal structure of HA has a shielding effect on the mucous membrane of the stomach and gut, thus, the absorption of toxic metabolites is reduced or fully prevented, which may ultimately result in better growth performance. Our results are contradictory to the findings of Celik et al [19] and Kaya et al [20] who reported no effect of humates supplementation on weight gain in broilers. These contrasting results of the effect of HA on weight gain might be related to the inclusion of lower levels of HA in the experimental diets, which were insufficient to affect growth.

The dietary addition of 5 g BC powder/kg diet improved (p<0.05 or 0.01) final weight, body weight and FCR but declined (p = 0.022) values of feed intake. The BC seed is widely used as a growth promoter. Abd El-Hack et al [6] stated that herbs are good for reducing microbial populations and also decrease the degradation of amino acids; hence, an improvement in weight gain might be observed due to the increased availability of amino acids. In contrast, Ali et al [21] observed no effect of BC on protein deposition and weight gain in broilers.

As shown in Table 2, it is obvious that dietary interaction between HA and BC, excluding 1.5 g HA×0 g BC, decreased (p<0.01) the total amount of consumed feed compared to the diet free of HA and BC. The lowest value of consumed feed was observed in the experimental group fed the 1.5 g HA×5 g BC. Similarly, Hakan et al [22] reported decreased feed intake in poultry birds fed HA supplemented diets. Attia et al [23] reported that feed intake was reduced by increasing levels of BC in laying quail feed. The decrease in feed intake might be related to higher availability and absorption of nutrients at the intestinal level and birds eating for the satisfaction of energy needs [24]; therefore, better utilization of feed caused early satisfaction of energy requirements. Better nutrient absorption might occur due to an increase in the mass of intestinal epithelial cells leading to improved absorption [3]. Conversely, Edmonds et al [25] concluded that supplementation of HA and humic substances in diets resulted in significant improvement in feed intake. Sogut et al [26] observed that supplementation of 3% and 5% BC seed in the feed of broilers improved feed intake. In these studies, the level of BC used was much higher than the level used in the present study (0.5%), thus lower levels of BC might not be able to impart changes in feed palatability.

Similar to final body weight and body weight gain, FCR tended to be improved with increasing HA levels and BC addition separately or combined. The best FCR was observed in birds that received the 2.25 g HA×5 g BC diet, whereas the poorest FCR was observed in birds fed unsupplemented diet. The HA might stabilize the flora in the intestinal tract and improve nutrient utilization. Therefore, an increase in live weight without increasing the amount of feed supplied to the birds resulted in better FCR [10]. In contrast, Kaya et al [20] postulated that dietary humates supplementation and other organic acids along with probiotics did not improve FCR. Previous studies have shown positive impacts of BC on broiler performance and improved FCR [27]. Abd El-Hack et al [6] also demonstrated that herbal plants improved the digestibility and absorption of amino acids. It was stated that herbal plants improved pancreatic secretion and digestive enzymes, which enhance absorption.

### Carcass characteristics

Results in Table 3 summarize the impact of dietary HA supplementation and BC addition and their interactions on carcass traits and visceral organ weights. Only carcass weight, breast yield, intestinal length, and intestinal weight were increased (p<0.05 or 0.01) by the dietary single additions of HA and BC or in combination. The highest level of HA (2.25 g/kg diet) recorded the best values of carcass weight, breast yield, intestinal length, and intestinal weight comparing with the control and other HA levels. Our results are in agreement with those reported by Aksu and Bozkurt [28] who found that breast and thigh weights were increased by the supplementation of HA in broiler diets. Results from the present study are contradictory with the findings of Avci et al [29] who reported that the breast weight of broilers was not affected by supplementation of HA and humates.

Enriching quail diet with 5 g BC powder/kg diet resulted in heavier (p<0.05) carcass, breast, and intestine than the diet free of BC (Table 3). Similar results were found by Abaza et al [30] who discovered that the addition of BC powder in broiler feed significantly improved carcass characteristics. Abd El-Hack et al [6] theorized that herbal plants have a good dietary effect on protein metabolism and nutrient utilization, which may lead to higher breast percentages. On the contrary, Abbas and Ahmed [31] also observed that the addition of 1% BC seed in broiler feed decreased dressing percentages. Al-Beitawi and El-Ghousein [32] and Ismail [33] stated that supplementing the diets with various levels of crushed or uncrushed BC did not influence any of the carcass traits in broilers. For the interaction between HA and BC, the highest values of carcass weight, breast yield, intestinal length, and intestinal weight were found in the 2.25 g HA×5 g BC group. Percentages of all visceral organs (intestine, liver, gizzard, heart, spleen, and bursa) were not statistically affected by dietary treatments.

### Gut microbial count

Results of microbial counts are, by any standards, unique (Table 4). Total viable microbial counts decreased (p<0.05) with increasing levels of HA except the intermediate level (1.5 g/kg diet). Consistent with our findings, Arif et al [2] reported that HA has a nutraceutical activity in that it stimulated neutrophil activity, which may provide protection against bacterial pathogens and reduce mortality during acute bacterial infection. Awwad et al [34] reported that HA affected most of the bacterial populations in the ceca of birds. Saki et al [35] reported that the use of organic acids reduced the total bacterial count in the gastrointestinal tract (GIT) through decreasing its pH.

The total coliform counts were significantly (p<0.05 or 0.01) lower in quail fed diet supplemented with 5 BC powder g/kg than those fed BC free diet. Ebru et al [36] stated that BC produced bactericidal secretions and decreased intestinal pH, which resulted in the reduction in the total counts of bacteria (*E. coli* and *Salmonella*) in quails. Ismail [33] reported that BC addition in quail feed reduced the total bacterial count and also reduced the quantities of *E. coli* and *Clostridium perfringens* in the GIT of quail. Attachment of type-1 fimbriae is one of the essential mechanisms for binding to the epithelium surface [37]. In this respect, it has been reported that BC oil serve as the attachment positions for Gram-negative bacteria, preventing their attachment onto the enterocytes as reported by Wielen et al [38]. This is the probable mechanism by which BC oil could decrease *Salmonella* and *E. coli* population in the current study. The BC seed oil was found to be rich in phenolics as well as other bioactive compounds that could serve as potential antimicrobial agents as postulated by Hassanien et al [39]. *In vitro* studies showed that a wide range of essential oils such as carvone might inhibit the growth of Gram-negative bacteria such as *E. coli* [40]. The effects of essential oils on growth inhibition of Gram-negative bacteria are comparable to antibiotics [41]. The results from literature indicated that no significant effects of essential oils on beneficial bacterial population in the gut of broiler chickens [41].

In the present study, the combined effect of HA and BC resulted in decreased microbial counts in quail (Table 4). The lowest total viable microbial count was found in the group of 2.25 g HA×5 g BC.

### Blood serum chemistry

Data presented in Table 5 shows no significant effects for HA levels, BC addition or the interaction between them on any of the determined blood parameters excluding cholesterol, HDL, and LDL. The concentration of serum cholesterol and LDL (excluding that 0.75 g HA) decreased (p<0.05) and HDL increased (p = 0.034) along with increasing HA level. Reduction of blood cholesterol and LDL may be due to decreasing the intracellular pH [42,43]. Our results from the present study are in agreement with the findings of Šamudovská and Demeterová [18]. Conversely, Kemal et al [44] reported that HA significantly influenced blood calcium, cholesterol, and phosphorus. Also, Rath et al [45] revealed that blood calcium, phosphorus were decreased by dietary HA supplementation.

Significant decrease (p = 0.044) in serum cholesterol and increase (p = 0.049) in serum HDL were detected as a response to BC supplementation in comparison to unsupplemented diet (Table 5). In line with our findings, Ali et al [21] noticed that 0.50% BC in feed decreased the level of blood LDL. However, they also observed that increasing BC up to 0.75% increased HDL levels. Our results are in contrast with the findings of Lotosh [46] who stated that glucose levels significantly increased by the addition of BC in broiler diet. Variation in reports might be related to differences in age, and the type and strains of experimental birds used [47].

## CONCLUSION

The aforementioned findings showed that HA and BC seed supplementation individually or combined improved growth performance of quails. Additives also improved gut health and decreasing microbial counts. Therefore, the combination of HA and BC seed could be an effective and beneficial growth promoter, with the recommended level being 2.25 g HA+5 g BC/kg of growing quail diet.

## IMPLICATIONS

Organic feed additives have attracted elevating attention as alternative to in-feed antibiotics in poultry production. These organic additives are more acceptable among consumers than chemical feed additives. The supplementation of BC and HA in quail diets improved growth performance and enhanced health status through decreasing microbial counts. The current paper concluded that the combination between HA and BC is a suitable feed additive for quail feeds with positive impact on quail growth performance.

## Figures and Tables

**Table 1:** Composition (as fed) and chemical analysis (on DM basis) of the experimental diet

Items	%
Ingredients	
Corn (8.5%)	53.03
Soybean meal (44%)	38.69
Gluten meal (62%)	3.20
Soybean oil	1.67
Di calcium phosphate	0.81
Limestone	0.30
Vit-min premix1)	0.30
NaCl	0.11
DL methionine (58%)	0.39
L-lysine HCl (119%)	1.50
Total	100
Chemical composition2)	
Crude protein	23.51
ME MJ/kg DM	12.06
Ca	0.85
P (available)	0.45
Lysine	1.60
Met.+Cys.	0.88
CF	3.92
Determined composition (%)3) on DM basis	
DM (% of the as-fed diet)	90.52
Crude protein	23.45
Crude fat	2.15
Ash	5.24
Crude fiber	3.56

DM, dry matter; ME, metabolizable energy; CF, crude fiber.

1) Growth vitamin and mineral premix each 2 kg consists of. Vit A 12,000,000 IU, Vit D_3_ 2,000,000 IU, Vit E 10 g, Vit K_3_ 2 g, Vit B_1_ 1,000 mg, Vit B_2_ 49 g, Vit B_6_ 105 g, Vit B_12_ 10 mg, pantothenic acid 10 g, niacin 20 g, folic acid 1,000 mg, biotin 50 g, choline chloride 500 mg, Fe 30 g, Mn 40 g, Cu 3 g, Co 200 mg, Si 100 mg, and Zn 45 g.

2) Calculated according to NRC [11].

3) According to AOAC [15].

**Table 2:** Effect of dietary treatments on growth performance of quail

Items		Initial weight (g)	Final weight (g)	Total feed intake (g)	Body weight gain (g)	FCR (g feed/g gain)
HA (g/kg diet)						
0		35.00	218.50^c^	502.84^a^	183.50^c^	2.74^a^
0.75		34.96	219.92^bc^	499.50^b^	184.96^bc^	2.70^b^
1.50		35.06	221.13^b^	479.30^c^	186.06^b^	2.58^c^
2.25		35.05	232.61^a^	458.65^d^	197.55^a^	2.32^d^
BC (g/kg diet)						
0		35.01	221.02^b^	492.25^a^	186.01^b^	2.65^a^
5		35.03	225.06^a^	477.89^b^	190.03^a^	2.52^b^
Interaction						
HA	BC					
0	0	35.06	217.12^e^	506.29^a^	182.06^e^	2.78^a^
	5	34.94	219.88^d^	499.38^b^	184.94^d^	2.70^b^
0.75	0	34.89	219.78^d^	499.29^b^	184.89^d^	2.70^b^
	5	35.03	220.06^a^	499.71^b^	185.03^d^	2.70^b^
1.5	0	35.17	217.34^e^	506.58^a^	182.17^e^	2.78^a^
	5	34.95	224.91^c^	452.02^d^	189.95^c^	2.38^c^
2.25	0	34.91	229.82^a^	456.83^c^	194.91^b^	2.34^d^
	5	35.19	235.40^a^	460.46^c^	200.18^a^	2.30^e^
SEM		0.23	0.46	1.05	0.23	0.01
Probabilities						
HA		0.582	0.025	0.011	0.030	<0.001
BC		0.845	0.012	0.022	0.021	<0.001
H×BC		0.916	<0.001	<0.001	0.012	<0.001

FCR, feed conversion ratio; HA, humic acid; BC, black cumin; SEM, standard error mean.

a–e Means in the same column with different superscripts differ (p<0.05).

**Table 3:** Effect of dietary treatments on carcass characteristics and visceral organs (% of slaughter weight) of quail

Items (% of slaughter weight)		Carcass	Breast	Leg quarter	Intestinal length (cm)	Intestine	Liver	Gizzard	Heart	Spleen	Bursa
HA (g/kg diet)											
0		64.24^b^	40.82^b^	23.19	70.55^bc^	4.44^c^	2.31	1.58	0.897	0.084	0.093
0.75		64.12^b^	40.77^b^	23.13	71.30^b^	4.46^c^	2.26	1.57	0.887	0.083	0.092
1.50		64.54^b^	41.56^a^	22.98	71.00^c^	4.58^b^	2.28	1.56	0.882	0.082	0.094
2.25		65.01^a^	41.95^a^	23.06	74.20^a^	5.04^a^	2.20	1.50	0.845	0.081	0.089
BC (g/kg diet)											
0		64.39^b^	41.15^b^	23.13	71.05^a^	4.55^b^	2.31	1.56	0.888	0.083	0.092
5		64.56^a^	41.40^a^	23.05	72.48^b^	4.71^a^	2.22	1.54	0.867	0.082	0.092
Interaction											
HA	BC										
0	0	64.34^ab^	41.08^b^	23.26	69.80^e^	4.42^b^	2.30	1.59	0.907	0.084	0.093
	5	64.13^ab^	40.56^c^	23.11	71.30^d^	4.46^b^	2.32	1.57	0.887	0.083	0.092
0.75	0	64.25^ab^	40.63^c^	23.16	71.30^d^	4.47^b^	2.28	1.57	0.887	0.083	0.092
	5	63.99^b^	40.90^bc^	23.09	71.30^d^	4.45^b^	2.23	1.57	0.886	0.083	0.092
1.5	0	64.14^ab^	41.08^b^	23.05	70.00^e^	4.41^b^	2.38	1.58	0.902	0.079	0.093
	5	64.94^ab^	42.04^a^	22.90	72.00^c^	4.74^b^	2.18	1.54	0.862	0.085	0.094
2.25	0	64.83^ab^	41.80^b^	23.03	73.10^b^	4.89^ab^	2.26	1.51	0.857	0.084	0.088
	5	65.18^a^	42.09^a^	23.09	75.30^a^	5.19^a^	2.13	1.48	0.832	0.077	0.090
SEM		2.09	1.78	1.03	0.92	0.33	0.15	0.14	0.08	0.02	0.02
Probabilities											
HA		0.041	0.035	0.354	0.005	0.012	0.365	0.652	0.325	0.953	0.321
BC		0.048	0.042	0.513	0.021	0.032	0.654	0.953	0.213	0.214	0.624
HA×BC		0.030	0.011	0.272	0.011	<0.001	0.994	0.997	0.982	0.996	0.996

HA, humic acid; BC, black cumin; SEM, standard error mean.

a–e Means in the same column with different superscripts differ (p<0.05).

**Table 4:** Effect of dietary treatments on intestinal microbial count of quail

Items (log 10/cfu)		Coliforms	*Escherichia coli*	*Clostridium perfringens*	pH
HA (g/kg diet)					
0		9.33^a^	8.00^a^	5.07^a^	6.71^a^
0.75		8.12^b^	7.10^b^	4.16^b^	6.51^b^
1.50		9.34^a^	8.00^a^	4.07^b^	6.70^a^
2.25		5.97^c^	5.08^c^	3.62^c^	5.99^c^
BC (g/kg diet)					
0		8.82^a^	7.49^a^	5.31^a^	6.57^a^
5		7.56^b^	6.60^b^	3.15^b^	6.38^b^
Interaction					
HA	BC				
0	0	10.55^a^	8.89^a^	5.98^a^	6.90^a^
	5	8.11^b^	7.11^b^	4.16^b^	6.51^b^
0.75	0	8.13^b^	7.10^b^	4.15^b^	6.51^b^
	5	8.16^b^	7.11^b^	4.16^c^	6.51^b^
1.5	0	10.55^a^	8.89^a^	5.99^a^	6.90^a^
	5	8.12^b^	7.10^b^	2.15^d^	6.49^b^
2.25	0	6.04^c^	5.08^c^	5.10^b^	5.98^c^
	5	5.89^c^	5.07^c^	2.13^d^	5.99^c^
SEM	0.033	0.032	0.032	0.02	
Probabilities					
HA		<0.001	<0.001	0.031	0.014
BC		0.032	<0.001	0.021	0.034
HA×BC		<0.001	<0.001	<0.001	<0.001

HA, humic acid; BC, black cumin; SEM, standard error mean.

a–d Means in the same column with different superscripts differ (p<0.05).

**Table 5:** Effect of dietary treatments on blood serum chemistry of quail

Items		ALP (μ/L)	ALT (μ/L)	AST (μ/L)	GPx (U/L)	SOD (U/L)	Cholesterol (mg/dL)	Triglyceride (g/dL)	Blood glucose	HDL (mg/dL)	LDL (mg/dL)	Calcium (mg/dL)	Phosphorus (mg/dL)
HA (g/kg diet)													
0		1,051.15	36.40	117.35	204.63	170.63	142.15^a^	110.03	97.70	110.93^c^	19.67^b^	11.03	5.09
0.75		1,050.75	36.01	117.01	205.78	171.38	141.60^b^	110.33	97.42	111.70^b^	20.92^a^	11.05	5.07
1.50		1,049.40	33.97	114.24	210.10	174.70	140.11^c^	110.43	97.97	111.75^ab^	19.48^b^	11.05	4.97
2.25		1,049.10	33.77	114.25	212.20	176.80	139.31^d^	110.28	97.24	112.84^a^	18.18^c^	10.98	5.11
BC (g/kg diet)													
0		1,050.03	34.99	115.64	208.04	173.14	140.86^a^	110.28	97.71	111.67^b^	19.60	11.05	5.05
5		1,050.18	35.09	115.78	208.31	173.61	140.73^b^	110.25	97.46	111.94^a^	19.53	11.00	5.07
Interaction													
HA	BC												
0	0	1,051.20	36.45	117.38	204.55	170.55	142.10^a^	109.93	97.61	110.81^d^	18.27^b^	11.06	5.10
	5	1,051.10	36.35	117.32	204.70	170.70	142.20^a^	110.13	97.79	111.04^c^	21.07^a^	11.00	5.07
0.75	0	1,050.30	35.56	116.53	206.60	171.80	141.10^ab^	110.73	97.63	112.24^b^	20.95^a^	11.08	5.09
	5	1,051.20	36.46	117.49	204.95	170.95	142.10^a^	109.92	97.20	111.16^c^	20.88^a^	11.01	5.05
1.5	0	1,049.40	33.97	114.26	210.10	174.70	140.11^b^	110.13	98.31	111.27^c^	20.68^a^	11.08	4.86
	5	1,049.40	33.96	114.22	210.10	174.70	140.10^b^	110.72	97.63	112.22^b^	18.28^b^	11.01	5.08
2.25	0	1,049.20	33.97	114.39	210.90	175.50	140.12^b^	110.33	97.27	112.35^b^	18.48^b^	10.98	5.15
	5	1,049.00	33.57	114.10	213.50	178.10	138.50^c^	110.23	97.20	113.32^a^	17.88^c^	10.98	5.06
SEM		0.37	0.35	0.34	0.84	0.85	0.42	0.45	0.66	0.55	0.46	0.06	0.09
Probabilities													
HA		0.542	0.651	0.615	0.315	0.984	0.022	0.652	0.652	0.034	0.032	0.651	0.954
BC		0.651	0.846	0.236	0.213	0.513	0.044	0.751	0.465	0.049	0.063	0.745	0.843
HA×BC		0.544	0.335	0.291	0.085	0.164	0.035	0.605	0.884	0.035	0.049	0.654	0.478

ALP, alkaline phosphate; ALT, alanine amino transferase; AST, aspartate amino transferase; GPx, glutathione peroxidase; SOD, superoxide dismutase; HDL, high density lipoprotein; LDL, low density lipoprotein; HA, humic acid; BC, black cumin; SEM, standard error mean.

a–d Means in the same column with different superscripts differ (p<0.05).
